# Oxidation of Amorphous Porous VO_x_ at Low Temperatures for the Formation of Thermochromic VO_2_ Films

**DOI:** 10.3390/nano16020130

**Published:** 2026-01-19

**Authors:** Hiedra Acosta-Rivera, Victor Rico, Francisco Javier Ferrer, Teresa Cristina Rojas, Rafael Alvarez, Nicolas Martin, Agustín R. González-Elipe, Alberto Palmero

**Affiliations:** 1 Instituto de Ciencia de Materiales de Sevilla, Consejo Superior de Investigaciones Científicas—Universidad de Sevilla (CSIC-US), Américo Vespucio 49, E-41092 Seville, Spain; 2 Centro Nacional de Aceleradores, Consejo Superior de Investigaciones Científicas—Universidad de Sevilla (CSIC-US), Thomas A. Edison 7, E-41092 Seville, Spain; 3 Departamento de Física Atómica, Molecular y Nuclear, Universidad de Sevilla, Aptdo 1065, E-41012 Seville, Spain; 4 Departamento de Física Aplicada I, Escuela Politécnica Superior, Universidad de Sevilla, Virgen de África 7, E-41011 Seville, Spain; 5 Université Marie et Louis Pasteur, SUPMICROTECH, Centre National de la Recherche Scientifique (CNRS), Institut FEMTO-ST, F-25000 Besançon, France

**Keywords:** thermochromic films, porous films, magnetron sputtering, oblique angle deposition, nanocolumns

## Abstract

Thermochromic VO_2_ crystalline domains have been formed in amorphous nanocolumnar VO_x_ films by means of a low-temperature oxidation process. The oxidation of an amorphous film with [O]/[V] below 1.9 favors the formation of VO_2_, V_3_O_7_, and V_2_O_5_ crystalline domains in the material for temperatures as low as 260 °C, while values above 1.9 lead to the sole formation of the V_2_O_5_ phase. It is found that the absorption of oxygen also causes a relevant film volume expansion that makes pores shrink. Under some specific conditions, low-temperature oxidation causes the near disappearance of the amorphous regions, clearly improving the overall transparency and optimizing the optical and electrical modulation capabilities associated with the presence of crystalline VO_2_ domains. The best thermochromic performance was found when the original stoichiometry was [O]/[V] = 1.5 and the oxidation temperature was 280 °C. These conditions yield a relatively transparent coating in the visible range that presents an optical modulation in the near-infrared range of nearly 50% and a drop of electrical resistivity of more than two orders of magnitude, with a transition temperature of 50.3 °C. A tentative model based on the volume expansion experienced by the film upon oxidation is proposed, which links the structural/chemical features of the material and the formation of the crystalline domains at such relatively low temperatures.

## 1. Introduction

VO_2_ is a well-known material that undergoes a Metal-Insulator Transition (MIT) at near room temperature, nominally at $T_{f}$ = 68 °C, which induces a shift in its crystalline structure from monoclinic to rutile and causes a drastic change in its electrical properties [1,2,3,4]. This feature has motivated the use of VO_2_ for numerous applications in different fields, e.g., as a channel layer in field-effect transistors (FETs), in memory devices, or in strain and gas sensor devices, among others (see, for instance, [5] and references therein). Additionally, the MIT causes a notable change in the optical transmittance of the material, particularly in the near-IR part of the spectrum, which also makes VO_2_ suitable for solar irradiation control applications in interior spaces and buildings, commonly known as “smart window” applications [6]. Here, the core functional principle relies on the ability to use a VO_2_-coated glazing to filter out near-IR light when the environmental temperature exceeds $T_{f}$, while maintaining a relatively high transparency in the visible range [1,7]. Since approximately 52% of sunlight at sea level falls within the IR region, such glazing would enable passive and indirect control of the interior temperature, reducing the use of alternative energy-intensive environmental control approaches, such as air conditioning or heating systems. Yet, the use of VO_2_ for smart window applications is currently hindered by several scientific and technical constraints. These include the need to lower $T_{f}$ to values closer to human comfort levels, ideally between 20 and 40 °C, as well as the optimization of the optical modulation in the near-IR region of the spectrum and of the transparency of the glazing in the visible range [6,8]. While these issues are being actively studied in the literature [9,10], an additional critical constraint relies on the very fabrication method, as current procedures demand processing temperatures well above 500 °C to trigger the crystallization of VO_2_ in its monoclinic phase, thus discarding the use of numerous common temperature-sensitive materials to support the coating [11]. In this paper we focus on this last issue and study a basic and simple strategy to trigger the formation and crystallization of VO_2_ at temperatures below 300 °C in porous films.

Several strategies are currently being explored in the literature to achieve the formation of monoclinic VO_2_ in films at low temperatures. These typically rely on a multistep technique, where a V-O-containing film is first synthesized and, subsequently, exposed to an oxidizing or reductive atmosphere at relatively low temperatures under the influence of an additional excitation source, such as lasers or UV lights [11,12,13,14]. For instance, in ref. [11], VO_2_ films fabricated via the sol–gel technique were treated with an excimer laser, achieving laser-induced crystallization within 60 s of exposure. Moreover, in ref. [12], the annealing temperature was reduced to approximately 250 °C by combining deep ultraviolet irradiation with a carbon-free oxidizer to generate reactive radicals. Other multistep techniques have been employed to grow VO_2_, e.g., by combining the Atomic Layer Deposition (ALD) technique and annealing processes at 300 °C (see reference [15] and references therein), the aggregation of nanoparticles and a subsequent oxidation at a temperature of 450 °C [16], or by remote plasma polymerization at a temperature of 480 °C [17], among others. However, a distinct approach was followed in ref. [18], in which a very thin bilayer structure made of V and V_2_O_5_ was annealed in an oxygen atmosphere at temperatures below 300 °C. This procedure resulted in the formation of monoclinic VO_2_ crystallites with thermochromic activity, thus demonstrating that this treatment at such relatively low temperatures may trigger the crystallization of VO_2_. Following this line of reasoning, our group has recently demonstrated that the low-temperature oxidation of a highly porous and amorphous nanocolumnar VO_x_ film could also induce the formation of VO_2_ crystallites at temperatures as low as 270 °C [19]. This preliminary result opened the door to exploring different processing conditions that may yield a larger amount of VO_2_ at low temperatures, an optimization of experimental processing parameters that may allow its practical application. In this paper, we perform this study by analyzing the influence of the original film stoichiometry and oxidation temperature on the formation of crystalline VO_2_ domains. Moreover, the optical and electrical features of the obtained films have been characterized to provide clear insights into their applicability.

Currently, there are numerous works that analyze the features and properties of porous nanocolumnar films grown by Physical Vapor Deposition techniques, such as magnetron sputtering [20] or electron-beam-assisted evaporation [21]. Specifically, the so-called magnetron sputtering technique at oblique angle deposition (MS-OAD) has been developed in the last decade as a variation of the classical magnetron sputtering deposition technique, by which a low-pressure plasma is ignited in a vacuum reactor to sputter atoms from a solid target, which are subsequently deposited on a substrate, making the film grow [22]. In this way, the MS-OAD technique proposes the use of a particular geometrical arrangement in the vacuum reactor by which the substrate is tilted with respect to the target in order to induce the oblique incidence of the deposition species [23]. This results in the formation of tilted and randomly scattered nanocolumnar structures, approximately a hundred nanometers wide, separated by large accessible mesopores that extend from the top surface of the film to the very substrate. Moreover, these nanocolumnar structures present a certain degree of internal open porosity in the form of micropores which, being connected to the large intercolumnar mesopores, are accessible from the outside by pore throats with diameters below 2 nm [24]. The existence of all this porosity makes these films have rather low densities and large specific surfaces in contact with the outside, as required for numerous applications, e.g., in sensors, biomedicine, or plasmonics, among others [25,26,27,28,29,30,31,32,33,34]. These types of morphology have already been investigated for thermochromic applications in the literature, mainly because of their relatively low density and lower effective refractive index [35]. Yet, the existence of a large accessible pore network in the films may represent an even more interesting feature when exposing the film to an oxidative atmosphere, as the uptake of oxygen would not only be limited to the top surface of the film, as is expected in compact homogeneous films, but rather throughout the whole accessible pore structure. Based on this idea and the results obtained in ref. [19], in this work, the low-temperature oxidation of different nanocolumnar amorphous VO_x_ films has been systematically analyzed, aiming at maximizing the formation of monoclinic VO_2_ in the material. In addition to the structural and chemical characterization of the films, the MIT has also been studied by analyzing the optical and electrical modulation properties during the MIT. The obtained results not only demonstrate the feasibility of this methodology to fabricate thermochromic coatings with relatively good optical properties at temperatures below 300 °C, but also provide interesting clues for understanding the importance of porosity and its interplay with the oxidation process for the stabilization of VO_2_ crystalline domains.

## 2. Experimental Setup

### 2.1. Materials

VO_x_ films were deposited at room temperature on doped silicon (1 0 0) wafers (Topsil) diced into 1 × 1 cm^2^ pieces, on silica (Vidrasa, Barcelona, Spain) diced into 1 × 1 cm^2^ pieces, and on a (0001) 99.9% pure Al_2_O_3_ sapphire substrate with a 31 mm diameter and a surface roughness lower than 0.5 nm. For MS-OAD, a V target with a 3′ diameter and a 99.95% purity (Testbourne, Hampshire, England) was used. Ar and O_2_ were used as plasma gases, with 99.99995% purity (Air Liquide, Sevilla, Spain). Prior to the deposition, the substrates were cleaned by conventional procedures in an ultrasonic bath (acetone, isopropanol, and deionized water). Other reagents and solvents were purchased as reagent-grade and used without further purification.

### 2.2. Deposition and Oxidation of Nanocolumnar VO_x_ Films

**Figure 1:**
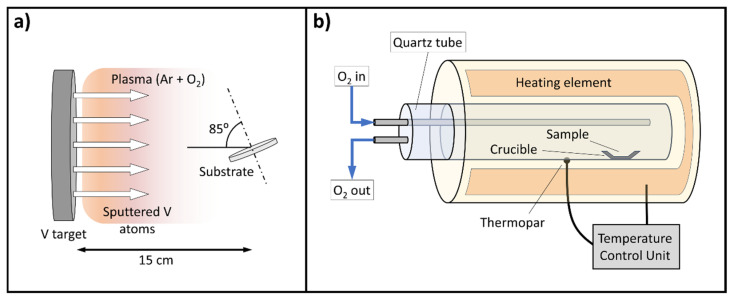
(**a**) Scheme of the magnetron sputtering reactor employed to grow the nanocolumnar films arranged according to the oblique angle configuration. (**b**) Scheme of the furnace employed to oxidize the coatings.

Nanocolumnar and amorphous films containing different amounts of O and V were deposited at room temperature by means of MS-OAD in a cylindrical deposition reactor that was 75 cm long and had an 18 cm radius, with a base pressure of 2 × 10^−4^ Pa (see scheme in Figure 1a). A 3-inch diameter V target was used as a cathode, with a substrate holder located 15 cm apart and aligned with the center of the target. An argon gas flow of 9.3 sccm was pumped into the reactor chamber along with a small flow of oxygen gas of 0.2 sccm that yielded a total pressure of 0.2 Pa. An electromagnetic power generator (Advanced Energy DC Pinnacle Plus, Littlehampton, UK) was employed to ignite a DC plasma, setting the value of the power between 150 W and 275 W. The substrate holder was tilted 85° with respect to the target’s surface, in a typical MS-OAD configuration, while the deposition time was set to obtain film thicknesses of around 300 nm. Table 1 contains the list of conditions employed to deposit the coatings studied in this work. A second set of depositions were carried out to grow compact films with different [O]/[V] ratios. In this case, a classic geometrical configuration was employed by placing the substrate holder parallel to the target’s surface, i.e., a substrate tilt of 0°, and using a power of 150 W (see deposition conditions in Table 1). In all cases, a pair of substrates consisting of a silicon wafer and a silica plate were utilized. For the electrical and Hall effect measurements, an additional set of sapphire substrates (dimensions: 3 × 3 cm) were used under the conditions described below.

After the deposition of the VO_x_ samples, they underwent an oxidation procedure in a furnace at atmospheric pressure using a temperature controller (EUROTHERM 2408, Worthing, UK) (see Figure 1b). Oxygen gas was fed into the furnace chamber, imposing a heating ramp of 5 °C min^−1^, up to a given target temperature that was kept constant during 90 min, after which the whole furnace was left to cool down until it reached room temperature. Here, it is important to mention that similar results as those presented in this paper regarding the thermochromic behavior of the films have also been obtained using atmospheric air instead of pure oxygen for the annealing process. Yet, under these conditions we cannot rule out that compounds other than oxides could be formed.

### 2.3. Characterization of Nanocolumnar VO_x_ Films

The morphology of the films was characterized by means of Scanning Electron Microscopy (SEM) with a high-resolution field-emission gun microscope model (HITACHI-S4800, Tokyo, Japan). The microstructure of the layers was also studied by Scanning Transmission Electron Microscopy (STEM): cross-sectional slices of the films deposited on silicon substrates were prepared using the conventional procedure of mechanical polishing followed by argon ion milling to electron transparency. High-angle annular dark-field STEM (HAADF-STEM) micrographs were acquired in a Tecnai G2 F30 S-Twin STEM (FEI, Eindhoven, The Netherlands), equipped with a HAADF detector (Fischione, PA, USA) with a 0.16 nm point resolution.

The [O]/[V] atomic concentration ratio in the films was determined by means of the Rutherford Backscattering Spectroscopy (RBS) and Nuclear Reaction Analysis (NRA) techniques. The setup employed to carry out these experiments was a 3 MV tandem Accelerator at the Centro Nacional de Aceleradores (CNA, Seville, Spain), employing a beam of 2.0 MeV alpha particles for RBS and a 0.9 MeV deuteron beam for the NRA, with a 1 mm beam spot diameter and passivated implanted planar silicon detectors, located at a 165° scattering angle for RBS and 150° for NRA. In the latter case, a 13 μm thickness filter of aluminized Mylar was placed in front of the detector to avoid scattered deuterons reaching the detector. The spectra have been analyzed using SIMNRA 6.0 code [36]. To ensure a consistent analysis, all reported measurements (morphological, chemical, electrical, and optical) have been performed at the central location of the substrate.

Raman spectroscopy analyses of the samples have been carried out using a LabRAM (Horiba Jobin Yvon, Glasgow, UK) with a green laser beam of 532 nm wavelength. An X-ray investigation was carried out by means of a D8 DISCOVER diffractometer (Bruker, Billerica, MA, USA). The device featured a 2D detector VANTEC-500 with a 2 mm capillary, which allowed us to obtain 2D frames corresponding to each point ranging from 10° to 60° 2θ. A copper Kα radiation source with a 0.15405 nm wavelength was used, with a step width of 20° for 1 h per step and tube conditions of 40 kV voltage and 40 mA current. Grazing Incidence X-ray Diffraction measurements were acquired by maintaining a fixed incidence angle of 0.5°.

The optical transmission properties of the films have been analyzed in the wavelength range of 200–2500 nm by means of an ultraviolet–visible spectrophotometer Lambda 750 S (PerkinElmer, Shelton, CT, USA) with a 60 mm diameter integrating sphere. In order to characterize the MIT, a homemade device consisting of two ceramic heaters with a pierced hole at the center was used to hold the samples during the ultraviolet–visible spectra acquisitions. The sample was heated up to 100 °C during optical characterization, with the device connected to a programmable power supply ISOTECH IPS-405 (RS pro, Madrid, Spain) operated in DC mode and using a thermocouple to read and control the temperature. Finally, the transmittance-versus-temperature thermochromic hysteresis loops were obtained employing the same setup, for a wavelength of 1500 nm in variable steps of temperature ranging from room temperature to 100 °C. DC electrical resistivity vs. temperature measurements of the furnace oxidized films were performed in a dark environment with a homemade setup. It involved a four-probe system with a Van der Pauw geometry in the temperature range of 25–100 °C with a ramp of 1 °C min^−1^ and then back to 25 °C with an equivalent negative ramp. Carrier mobility and carrier concentration were obtained by Hall effect measurements using the same Van der Pauw geometry. A constant magnetic field of 0.800 T was applied perpendicular to the sample surface.

In terms of notation and labeling of the samples, in this paper we have chosen the following criterion: as-deposited films have been labeled with the prefix “nano-” or “compact-” depending on whether the structure is nanocolumnar or compact, respectively, followed by “VO_x_”, with x being the measured value of the [O]/[V] ratio in the as-deposited film. For instance, nano-VO_1_._5_ refers to an as-deposited nanocolumnar film with a measured value of [O]/[V] = 1.5. Moreover, the films that have undergone an oxidation process also include the value of the oxidation temperature in their label. For instance, the film nano-VO_1.5_ (280 °C) refers to a nanocolumnar film with an as-deposited stoichiometry of [O]/[V] = 1.5 that has been subjected to oxidation at a temperature of 280 °C (note that the value of the [O]/[V] ratio after the oxidation process is not included in the label).

## 3. Results and Discussion

### 3.1. Analysis of the Nanocolumnar VO_x_ Films Before and After the Oxidation Process

**Figure 2:**
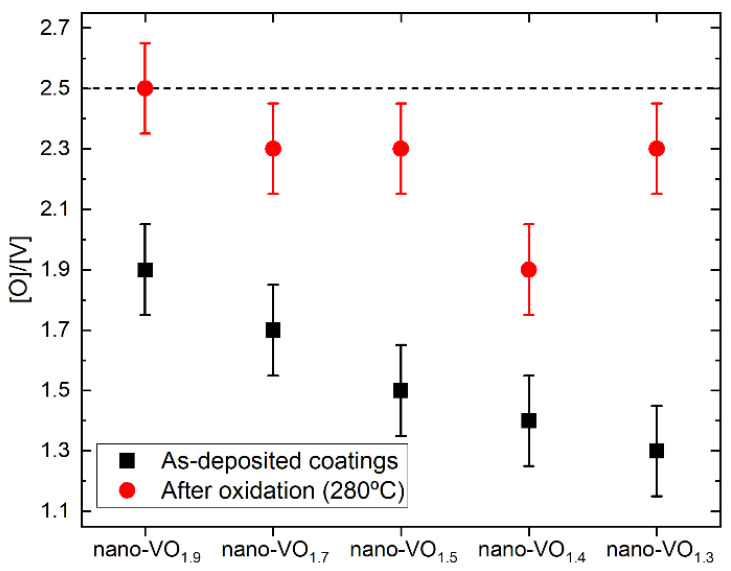
Measured values of the atomic concentration ratio [O]/[V] in each nanocolumnar film before and after the oxidation process at 280 °C.

**Figure 3:**
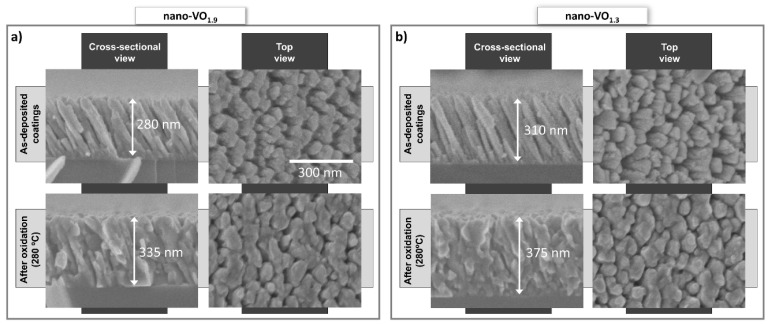
(**a**) Cross-sectional and top-view SEM images of the film nano-VO_1.9_ before and after oxidation at 280 °C. (**b**) Cross-sectional and top-view SEM image of the film nano-VO_1.3_ before and after oxidation at 280 °C.

As mentioned in the previous section, a set of nanocolumnar films were grown with different [O]/[V] ratios by means of the MS-OAD technique, by varying the value of the DC sputtering power from 150 W to 275 W and maintaining a constant flow of oxygen in the reactor (see Table 1). The measured value of the [O]/[V] ratio of these films is depicted in Figure 2, where a clear decreasing trend in the oxygen content with power is obtained, from [O]/[V](150 W) = 1.9 to [O]/[V](275 W) = 1.3 (the error of these values is estimated ±0.15 in all these cases). This systematic change can be explained by the increase in the sputtering rate of V atoms for increasing powers and, thus, in the deposition rate of V atoms. From a morphological point of view, the as-deposited films are alike, as illustrated in Figure 3a,b, where the cross-sectional FESEM images of nano-VO_1.9_ (Figure 3a, top left) and nano-VO_1.3_ (Figure 3b, top left) are shown. Interestingly, and despite the different chemical composition of both films, a similar columnar morphology is apparent in both cases: tilted ~30° with respect to the substrate normal, with column diameters of ~100 nm and separated by noticeable large intercolumnar mesopores. The origin of such similar morphology stems from the common geometrical oblique angle arrangement in both cases, which is known for mediating surface shadowing mechanisms and the subsequent formation of the tilted nanocolumnar structures. This is apparent in the top-view images of these films, also depicted in Figure 3a,b (top right images), where similar nanocolumnar and pore morphologies can be observed. Moreover, the absence of peaks in the Raman analyses, along with the high opacity of these coatings, indicate that they are mostly composed of oxygen-deficient amorphous V-O domains.

**Figure 4:**
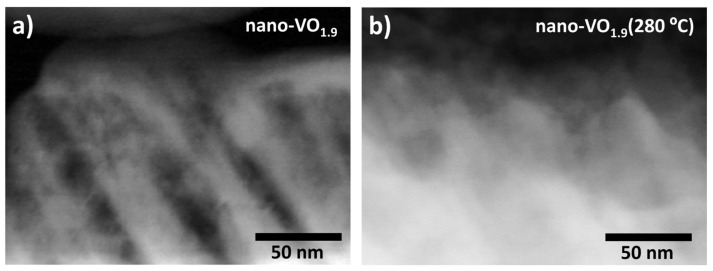
STEM-HAADF image of the tips of the nanocolumns in the film nano-VO_1.9_ before (**a**) and after (**b**) being subjected to the oxidation process at 280 °C.

The as-deposited films were placed in a furnace and exposed to an oxygen atmosphere at different temperatures, as described in the Experimental Setup section. After this oxidation process, the value of the [O]/[V] ratio in the films clearly increased. For illustration purposes, the measured values of [O]/[V] after oxidation at 280 °C are included in Figure 2. There, it is remarkable that the film nano-VO_1.9_ reaches the oxygen saturation value ([O]/[V] = 2.5) after oxidation, in agreement with the results in ref. [19], while the remaining samples present post-oxidation stoichiometries between 1.9 and 2.3. Moreover, from a morphological point of view, the oxidation process also causes important structural changes, which are illustrated in Figure 3a,b, where the top and cross-sectional FESEM views of the coatings nano-VO_1.9_ (280 °C) (Figure 3a, bottom images) and nano-VO_1.3_ (280 °C) (Figure 3b, bottom images) are displayed. There, it is noticeable that the oxidation has caused the widening of the nanocolumns up to the point of merging at some locations, shrinking the pores and forming a more compact structure. Furthermore, Figure 3 evidences an overall increase in thickness of about 20% in both cases, despite the fact that these images were taken from the same samples before and after the oxidation process and, approximately, at the same location (positioning error below 1 mm), finding a shift in thickness from 280 nm to 335 nm in the case of nano-VO_1.9_ and from 310 nm to 375 nm in the case nano-VO_1.3_. We attribute this swelling of the structure to the incorporation of O into the film network, which is known for increasing the associated volume per V atom in the film (a simple calculation based on the standard density of different vanadium oxide materials results in increasing values of volume per V atom in the material, from $\sim 1.4\times 10^{-2}\;{nm}^{3}/V\;atom$ in pure V to $\sim 4.5\times 10^{-2}\;{nm}^{3}/V\;atom$ in V_2_O_5_). This swelling phenomenon would additionally be favored by the relatively high mobility of the species in the V_2_O_5_ domains at such low temperatures (as it will be demonstrated below, this phase appears in all these coatings after oxidation), with a Tamman temperature as low as ~200 °C (for comparison purposes, the Tamman temperature in VO_2_ amounts to ~847 °C). This swelling phenomenon is further confirmed in Figure 4, where HAADF-STEM images of the tips of the nanocolumns in nano-VO_1.9_ and nano-VO_1.9_ (280 °C) are displayed, and where it is clearly shown how the intercolumnar mesopores shrink and a more compact structure is formed after oxidation.

**Figure 5:**
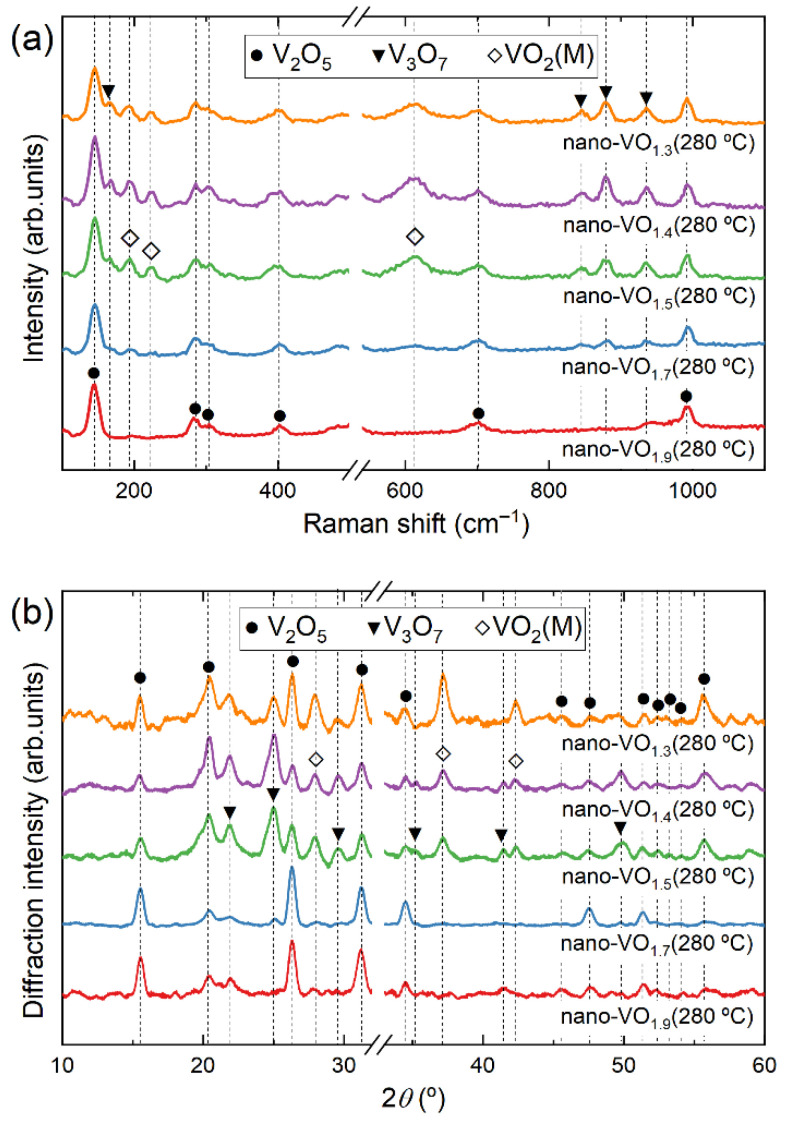
Raman (**a**) and XRD (**b**) analyses of the different nanocolumnar films after being subjected to the oxidation process at 280 °C.

**Figure 6:**
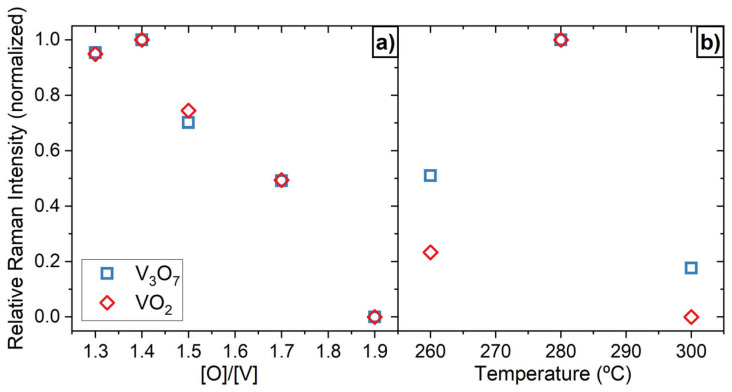
Relative intensity of peaks corresponding to VO_2_ (222 cm^−1^) and V_3_O_7_ (871 cm^−1^) phases with respect to that of V_2_O_5_ (700 cm^−1^) (**a**) taken from Figure 5a and (**b**) taken from Figure 7a.

In Figure 5a, the Raman spectra of the nanocolumnar films after oxidation at 280 °C are presented. There, it is apparent that nano-VO_1.9_ (280 °C) contains V_2_O_5_ crystalline domains, which is coherent with the measured value [O]/[V]~2.5 reported in Figure 2. Moreover, the spectrum of nano-VO_1.7_ (280 °C) indicates not only the existence of V_2_O_5_, but also of V_3_O_7_ and VO_2_ crystalline domains in the film. This becomes even more evident for samples nano-VO_1.5_ (280 °C), nano-VO_1.4_ (280 °C), and nano-VO_1.3_ (280 °C), where the peaks associated with VO_2_ are clearly discernable. Consequently, it has been found that whenever the as-deposited [O]/[V] ratio stays below 1.9, the oxidation process at 280 °C triggers the formation of VO_2_ along with the V_2_O_5_ and V_3_O_7_ phases. The appearance of these phases as a function of the original stoichiometry of the films is qualitatively assessed in Figure 6a, where the relative intensity of the peaks corresponding to VO_2_ (222 cm^−1^) and V_3_O_7_ (871 cm^−1^) phases with respect to that of V_2_O_5_ (700 cm^−1^) in Figure 5 are depicted. There, it is clear that both the VO_2_ and the V_3_O_7_ peaks evolve together, reaching their maximum value when the original stoichiometry is below 1.5, with a decreasing trend up to a stoichiometry of 1.9, when both peaks disappear. This is also corroborated by XRD analyses of the same films, presented in Figure 5b, where the peaks corresponding to monoclinic VO_2_, V_2_O_5_, and V_3_O_7_ phases are evident.

**Figure 7:**
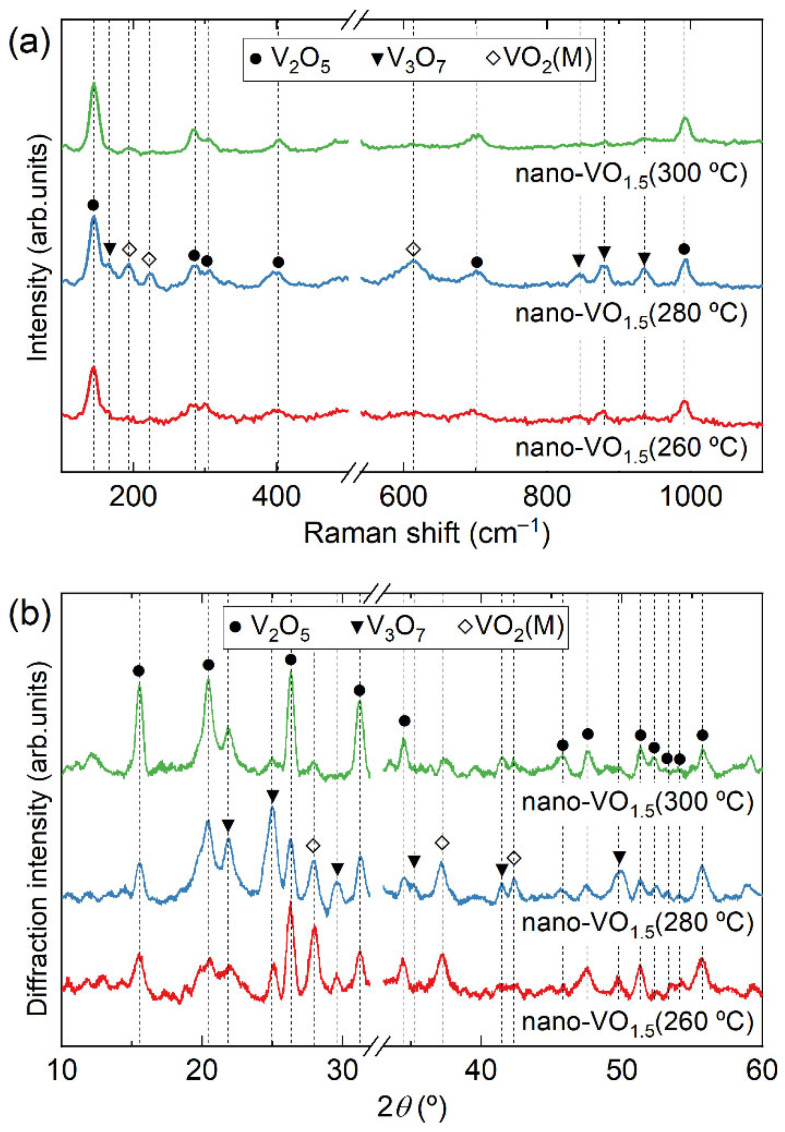
Raman (**a**) and XRD (**b**) analyses of the film nano-VO_1.5_ after being subjected to the oxidation process at temperatures of 260 °C, 280 °C, and 300 °C.

In addition to the [O]/[V] ratio in the original (as-deposited) films, the oxidation temperature also plays a key role in the formation of VO_2_ crystalline domains. Figure 7a,b shows the Raman spectra and the XRD patterns of the film nano-VO_1.5_ after being subjected to an oxidation procedure at temperatures of 260 °C, 280 °C, and 300 °C, respectively. There, the peaks associated with V_2_O_5_ are evident in all cases, while the V_3_O_7_ and VO_2_ phases are clearly discernable in nano-VO_1.5_ (280 °C), as well as in nano-VO_1.5_ (260 °C) and nano-VO_1.5_ (300 °C), even though they are not as intense in these two latter cases. This implies that nano-VO_1.5_ (280 °C) seems to represent the optimum case in terms of VO_2_ peak intensity. This is corroborated in Figure 6b, in which the relative intensity of the peaks of the Raman spectra corresponding to the VO_2_ (222 cm^−1^) and V_3_O_7_ (871 cm^−1^) phases with respect to that of V_2_O_5_ (700 cm^−1^), taken from Figure 5, are shown, and where it is clear that the optimum oxidation temperature is 280 °C. According to Figure 5, Figure 6 and Figure 7, it is remarkable that VO_2_ in these coatings always coexists with the V_2_O_5_ and the V_3_O_7_ phases, which provides some clues to how the oxygen incorporates into the film network and induces the formation of different crystalline domains, with V_2_O_5_ being the only crystalline phase that has been found isolated from the other two. It is noteworthy in this regard that the crystal size, determined by the Scherrer equation [37] from the width of the peaks in Figure 5b, renders approximate values of ~10 nm for VO_2_, ~15 nm for V_3_O_7_, and ~25 nm for V_2_O_5_. In Section 3.3, a model of the formation of VO_2_ at low temperatures that takes into account the effect of film stoichiometry, porosity, and oxidation temperature on the formation of VO_2_ is presented.

### 3.2. Optical and Thermochromic Properties

**Figure 8:**
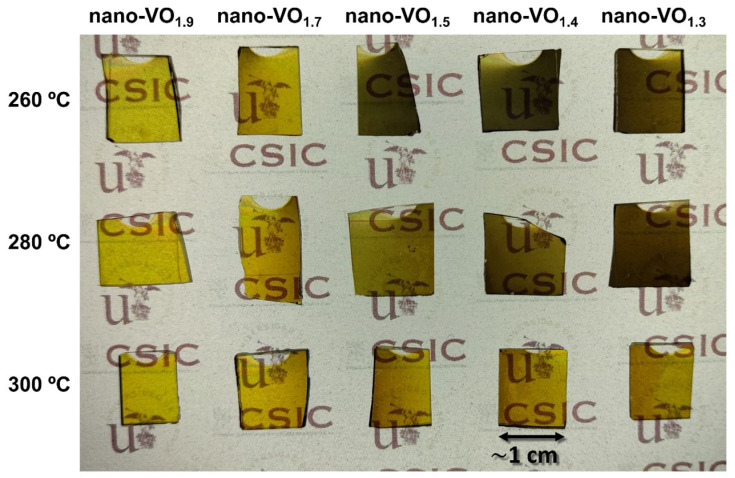
Photograph of the nanocolumnar films grown on SiO_2_ with as-deposited stoichiometry ranging from 1.3 to 1.9 after being subjected to the oxidation process at temperatures of 260 °C, 280 °C, and 300 °C.

Figure 8 presents a photograph of the samples deposited on SiO_2_ substrates after the oxidation process at different temperatures. These films present a brownish color, which is typical of V-O films, and show certain transparency, especially the films nano-VO_1.9_ (260 °C, 280 °C, and 300 °C) and nano-VO_1.7_ (260 °C, 280 °C, and 300 °C), no matter the oxidation temperature. This agrees with the existence of a large amount of V_2_O_5_ crystalline domains in these cases, as obtained in the XRD/Raman analyses in Figure 5, Figure 6 and Figure 7. Interestingly, the transparency of the remaining samples is highly dependent on the oxidation temperature. They are slightly opaque when the temperature is 260 °C and increasingly transparent when the temperature is 280 °C and 300 °C, suggesting that a certain amount of (oxygen-deficient) amorphous VO_x_ regions survive the oxidation process, which are progressively removed as the oxidation temperature increases.

**Figure 9:**
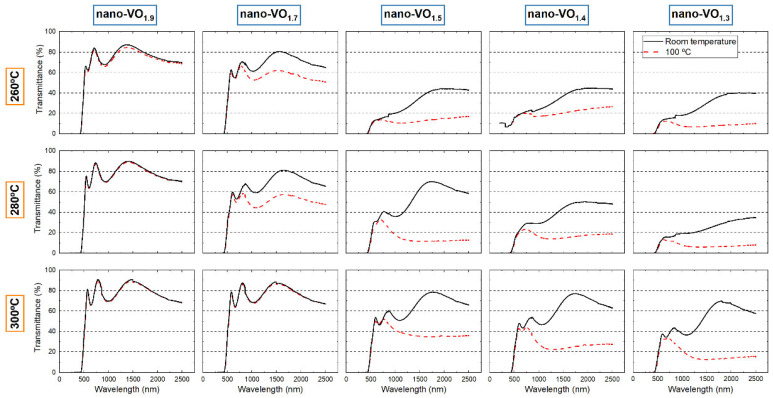
Optical transmittance spectra of the nanocolumnar coatings after being subjected to the oxidation process at 260 °C, 280 °C, and 300 °C. Results measured at room temperature (black) and at 100 °C (red) are included.

In Figure 9, the vis-IR spectra of the films depicted in Figure 8 are presented, when measured both at room temperature and at 100 °C. As expected, the films nano-VO_1.9_ (260 °C), nano-VO_1.9_ (280 °C), and nano-VO_1.9_ (300 °C) show typical spectra of V_2_O_5_ as well as no thermochromic behavior. This agrees with the XRD/Raman analyses in Figure 5, Figure 6 and Figure 7, and with the lack of any VO_2_ signal in these cases. Remarkably, sample nano-VO_1.5_ (280 °C) possesses a transmittance spectrum quite similar to that reported in the literature for (non-doped) VO_2_ films [38,39,40], characterized by a clear thermochromic transition in the IR region from ~60% at room temperature to ~10% at 100 °C, with a transmittance in the visible part of the spectrum of ~40% no matter the environmental temperature. This agrees with the Raman and XRD results in Figure 5, Figure 6 and Figure 7 and the relevant amount of VO_2_ crystal domains detected for these samples. For the rest of the cases, the optical modulation is coherent with the existence of VO_2_ crystalline domains, as measured in Figure 5, Figure 6 and Figure 7, along with amorphous oxygen-deficient VO_x_ that hinders the overall optical transparency of the coatings. In this regard, according to Figure 8, the amount of amorphous VO_x_ after oxidation seems to diminish for increasing values of the as-deposited [O]/[V] ratio and the oxidation temperature. This result indicates the delicate interplay between as-deposited nanocolumnar film stoichiometry and oxidation temperature, not only for the formation of the different crystalline phases, namely V_2_O_5_, V_3_O_7_, and VO_2_, but also for the removal of the oxygen-deficient amorphous VO_x_ regions, the latter having a strong influence on the overall transparency of the film.

**Figure 10:**
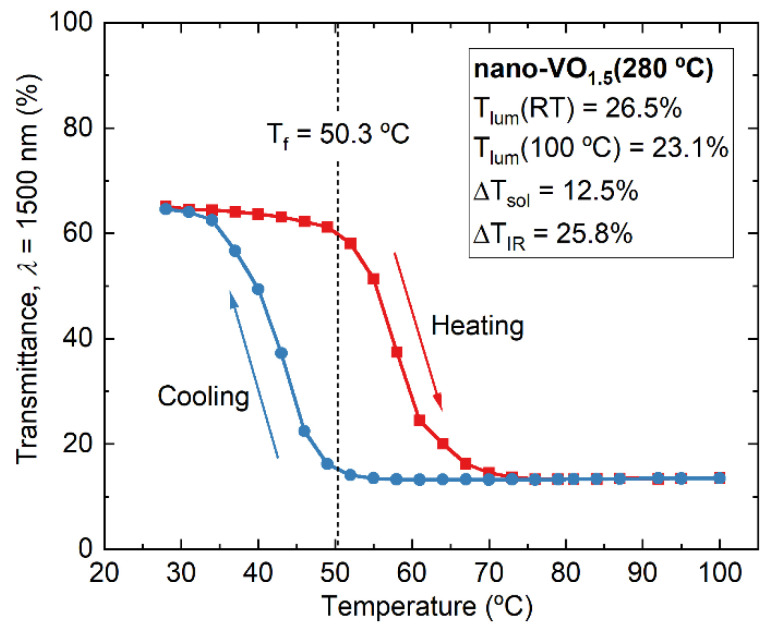
Hysteresis loop corresponding to the changes in the optical transmittance of the film nano-VO_1.5_ (280 °C) measured at a wavelength of 1500 nm as a function of the environmental temperature after a single heating/cooling cycle from 25 °C to 100 °C and back. The quantities $T_{lum},\;∆T_{sol}$, and $∆T_{IR}$ (see main text for their definition) are included along with the transition temperature (centroid of the loop curve), $T_{f}$.

**Figure 11:**
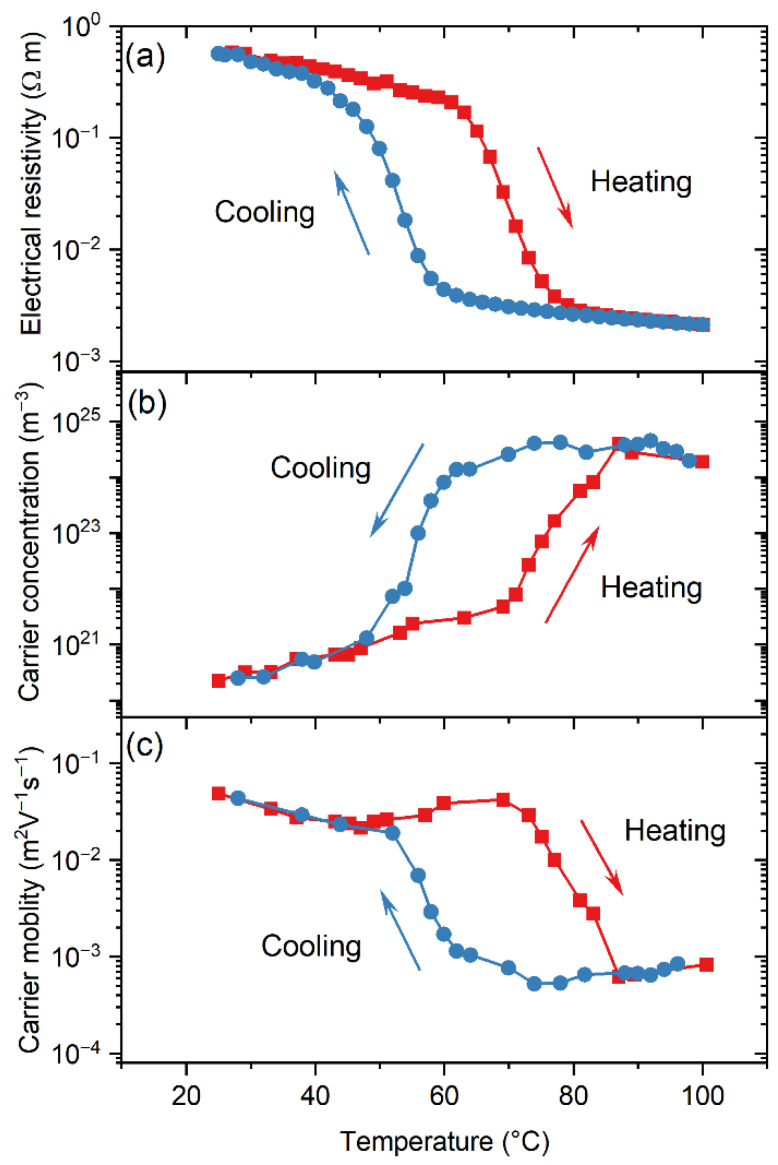
Electrical resistivity (**a**), carrier concentration (**b**), and mobility (**c**) as a function of the environmental temperature of the film nano-VO_1.5_ (280 °C). A single heating–cooling cycle was applied, which corresponds to a heating from 25 °C to 100 °C, then a cooling back to 25 °C.

Given the results presented in Figure 9, we have chosen the film nano-VO_1.5_ (280 °C) as the best case in terms of thermochromic efficiency; it was also chosen for further analysis of its optical and electrical modulation capabilities during the MIT. Figure 10 showcases the changes in transmittance at a wavelength of 1500 nm when varying the temperature of nano-VO_1.5_ (280 °C) from room temperature to 100 °C and back. A typical hysteresis loop corresponding to a MIT is reproduced, where a transmittance drop of ~50% (from ~65% to ~15%) is found, with the centroid of the hysteresis loop at a transition temperature of $T_{f}$ = 50.3 °C. To evaluate the thermochromic efficiency of this coating, different standard quantities have been calculated, such as $T_{lum}$ that quantifies the visible light transmitted by the coating and is used as an indicator of luminosity (see appendix I in ref. [19] for its definition). The value of this parameter is 26.5% (at room temperature) or 23.1% (at 100 °C). Moreover, the solar modulation, $∆T_{sol}$, has a value of 12.5%, taken as a reference of the radiation not transmitted by the film due to the thermochromic transition. This means that this coating possesses a high enough optical modulation to be employed for practical applications (the general consensus for this matter is $∆T_{sol}>10\%$ for a proper adaptation to different climate changes [41,42]). In this regard, the only constraint for its direct use is its poor optical transparency in the visible range; $T_{lum}=26.5\%$ at room temperature (please note that reported values for $T_{lum}$ for pure VO_2_ are about 50% [17,43]), which we attribute to the presence of oxygen-deficient amorphous VO_x_ in the films as well as the V_3_O_7_ phase. Actually, V_3_O_7_ is known for absorbing in the visible range [44,45], thus hindering the transparency of the film. Nevertheless, the value of $T_{lum}$ can be improved by means of well-established strategies, e.g., by using doping or laser techniques [39,46], that enable values of $T_{lum}\sim 60\%$ [47]. The electrical modulation features of nano-VO_1.5_ (280 °C) during the MIT were also assessed by means of the Van der Pauw method and Hall effect as a function of temperature (note that the substrate employed to support the coating for this study was sapphire, with a larger size than the silica substrate). This film underwent a significant temperature-induced transition during heating, where the DC electrical resistivity abruptly changed more than two orders of magnitude from 10^−1^ to 10^−3^ Ω·m when the temperature was varied from 25 to 100 °C (Figure 11a), a drop characteristic of VO_2_ compounds [48] yet smaller than that reported for pure VO_2_, which is usually of about four orders of magnitude (see for instance refs. [48,49]). In our case, we attribute this discrepancy to the existence of additional V_2_O_5_ and V_3_O_7_ crystalline phases in the nano-VO_1.5_ (280 °C) film, as demonstrated above when discussing Figure 5, Figure 6 and Figure 7, along oxygen-deficient amorphous VO_x_ regions. Interestingly, the measured hysteresis loop in the cooling part depicted a sharp increase in resistivity when the temperature reached ~53 °C, i.e., 15 °C below the nominal value of the transition at 68 °C for pure VO_2_ phases [50]. Overall, the features of the measured hysteresis curve, such as the abrupt changes in resistivity, support that the preparation conditions of nano-VO_1.5_ (280 °C) favor the formation of a significant amount of VO_2_ domains. Hall effect measurements on nano-VO_1.5_ (280 °C) show that electrons are the majority carriers irrespective of the temperature range, revealing the n-type nature of this film. Electron concentration and mobility vs. temperature also show strong variations and hysteresis loops, as shown in Figure 11b and Figure 11c, respectively. Upon increasing temperature, concentration increases from 2.3 × 10^19^ m^−3^ at 25 °C to 2.0 × 10^24^ m^−3^ at 100 °C. For these same temperatures, mobility exhibits a reverse evolution, reducing from 4.9 × 10^−2^ m^2^V^−1^s^−1^ to 8.3 × 10^−4^ m^2^V^−1^s^−1^, which shows that the drop of resistivity can be mainly assigned to the electron concentration change, in agreement with results in the literature [51,52,53,54]. During the heating stage and up to 70 °C, it is noteworthy that the concentration increases continuously with temperature (whereas mobility remains in the same order of magnitude, i.e., in the range of 2–5 × 10^−2^ m^2^V^−1^s^−1^, which defines a semiconducting-like behavior. Compared to optical transmittance vs. temperature measurements performed at a wavelength of 1500 nm (Figure 10), it is important to remark that the transition of electron transport properties occurs at a higher temperature, with a centroid of the resistivity hysteresis at about ~62 °C, which is significantly higher than that determined from the optical transmittance (50.3 °C). Such a difference has also been reported without discussing this effect [43,55,56,57]. One may suggest that the higher temperature values found for the resistivity hysteresis can be assigned to the difference in the interactions of the VO_2_ phase with electrons and photons. Optical transmittance is sensitive to changes in local dielectric properties, whereas resistivity depends on electrical current traveling through the different phases and grains and so on the distance between the electrodes. As a result, current represents a macroscopic probing of the transition and is less sensitive to mean local dielectric variations. However, this discrepancy can also be attributed to the different size and nature of the substrate employed for the electrical characterization or to small inhomogeneities in the coating that might affect the measurements.

### 3.3. Low-Temperature Formation of VO_2_ in Porous Nanocolumnar Films

The results above, in which small VO_2_ domains have been crystallized in their monoclinic phase at temperatures below 300 °C, contrast with the nominal value of crystallization temperature of VO_2_ in the bulk, which is well above 500 °C. This means that, in our case, the oxidation process must act as an excitation source that favors the low-temperature crystallization of VO_2_, as was also concluded in refs. [18,19]. Moreover, it has been demonstrated that the stoichiometry of the original (as-deposited) film and the oxidation temperature play key roles in the formation of the different crystalline phases, as well as in the survival of oxygen-deficient amorphous VO_x_ in the material after oxidation. Yet, the influence of film porosity has still not been specifically addressed, as this feature was rather similar in all the as-deposited films presented above. Keeping this purpose in mind, additional experiments have been carried out on a set of compact layers deposited using the classical magnetron sputtering arrangement under the conditions listed in Table 1. They were deposited with a similar thickness as the nanocolumnar ones (~300 nm) and with different [O]/[V] ratios, labeled as compact-V, compact-VO_0.4_, compact-VO_0.9_, and compact-VO_1.5_. The as-deposited [O]/[V] ratio in these films was measured using the same methodology (RBS/NRA), finding the values 0.0, 0.4, 0.9, and 1.5, respectively (the error of these values is estimated as ±0.15). After deposition, all of them were opaque, showed a compact nanostructure, and, attending to the absence of peaks in the Raman and the XRD analyses, did not show any sign of crystalline phases, indicating that, similarly to the as-deposited nanocolumnar films, they are formed by oxygen-deficient amorphous VO_x_.

**Figure 12:**
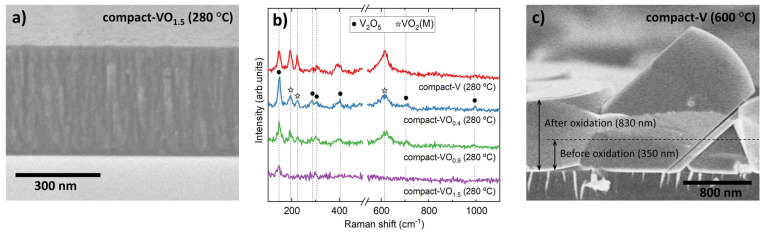
(**a**) Cross-sectional image of the film compact-VO_1.5_ (280 °C). (**b**) Raman analysis of the compact films after oxidation at a temperature of 280 °C. (**c**) Cross-sectional image of a compact metallic V thin film with an as-deposited thickness of 350 nm after being subjected to an oxidation process at 600 °C.

**Figure 13:**
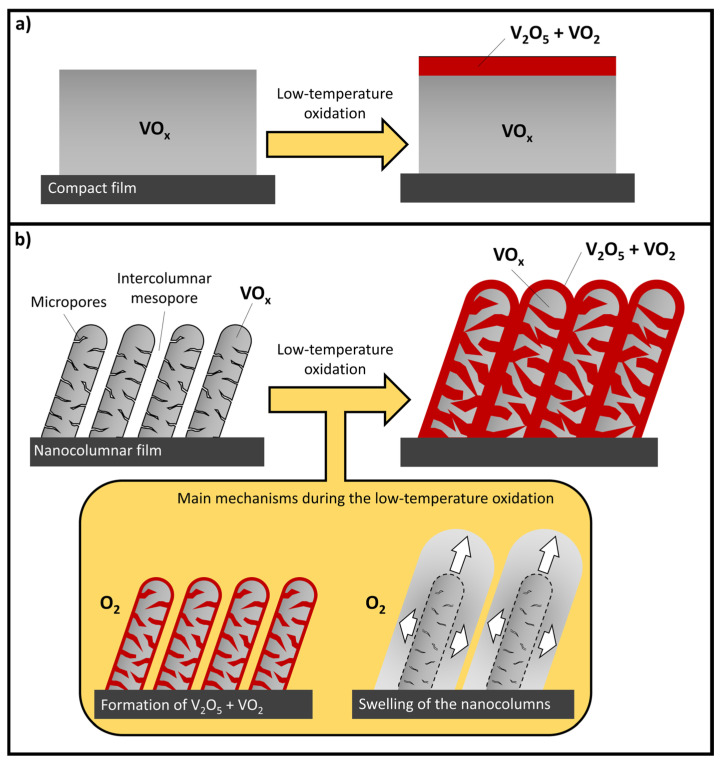
Scheme of the main mechanism during the low-temperature oxidation of the compact (**a**) and nanocolumnar (**b**) coatings. Note that the pores in the nanocolumnar case are magnified for clarity purposes.

These compact layers were subjected to the same oxidation process as the nanocolumnar ones at a temperature of 280 °C, finding that all of them still remained compact (as an example, the cross-sectional SEM image of compact-VO_1.5_ (280 °C) is depicted in Figure 12a) and opaque. This indicates that low-temperature oxidation is not efficient for these films and that a large amount of amorphous VO_x_ must still survive. However, the Raman analysis of these coatings (see Figure 12b) reveals the formation of V_2_O_5_ crystal domains in all cases, in agreement with the results obtained with the nanocolumnar films (see Figure 5 for instance). Remarkably, there are traces of VO_2_ in the samples compact-V (280 °C), compact-VO_0.4_ (280 °C), and compact-VO_0.9_ (280 °C), demonstrating that the proposed methodology also triggers the crystallization of the V_2_O_5_ and VO_2_ phases in compact films at low temperatures. Yet, there is no clear indication of any variation in film thickness associated with the oxidation process at 280 °C; this feature, along with the lack of transparency of these films, suggests that only a small portion of material on the film surface is affected by low-temperature oxidation, since the associated swelling phenomenon is restricted to this very shallow region. Remarkably, these two features (structural swelling and transparency) are evident whenever a compact film is efficiently oxidized. For instance, in Figure 12c, the cross-sectional SEM image of a pure V film with an original thickness of 350 nm, after being subjected to an oxidation process at a much higher temperature (600 °C), is presented for illustration purposes. In this case, the film was not only transparent after oxidation (with composition V_2_O_5_), but also showed clear indications of swelling, with an increase in film thickness from ~350 nm to ~830 nm that even caused the direct delamination of the coating at some locations. Based on this result, a basic scheme of the oxidation process of compact films at low temperatures is presented in Figure 13a: there, it is proposed that low-temperature oxidation only affects a specific shallow region in contact with the oxygen atmosphere, where the (highly mobile) V_2_O_5_ phase and the VO_2_ crystallites are formed, limiting the diffusion of oxygen towards the inside of the material, which remains unaffected.

Based on the idea presented in Figure 13a, we have also tentatively put forward a basic scheme that rationalizes the main results obtained in this paper on nanocolumnar porous films, which is shown in Figure 13b. There, the as-deposited film is depicted as an array of large and tilted amorphous VO_x_ nanocolumns separated by large intercolumnar mesopores that penetrate into the nanocolumns by means of large and elongated micropores (please note that the size of the pores is magnified for clarity purposes). In this way, when the film is exposed to the oxygen atmosphere at low temperatures, the whole surface of the nanocolumns (including that of the accessible pores) becomes oxidized, resulting in the formation of V_2_O_5_ and VO_2_, just like in the compact case (Figure 13a). Following an analogous reasoning, this oxide layer likely limits the diffusion of oxygen towards the inside of the nanocolumns, not affecting the VO_x_ material inside, in a process that is also enhanced by the swelling of the material and the shrinkage/disappearance of the mesopores and nanopores. Consequently, according to this scheme, the resulting film would contain not only oxygen-deficient amorphous VO_x_ inside the nanocolumns, but also crystallites of V_2_O_5_ and VO_2_. Therefore, when the original film stoichiometry is high enough (in our case when the as-deposited [O]/[V] ratio is above 1.9) or the oxidation temperature is 300 °C or above, it has been found that the oxidation process becomes efficient enough to oxidize the amorphous VO_x_ material in the nanocolumns and saturate the whole structure with oxygen, resulting in the formation of a pure V_2_O_5_ film. Additionally, it has also been found that there is an optimum value of the original film stoichiometry/oxidation temperature (in our case when [O]/[V]~1.5 and the oxidation temperature is 280 °C) at which the delicate balance between the adsorption of oxygen on the surface of the pores and the swelling of the material and the shrinkage of the pores, as well as the limitation of the oxygen diffusion towards the inside of the film, produces the removal of most amorphous VO_x_ while preserving the VO_2_ crystallites in the structure, which clearly improves the optical transparency of the coatings. Finally, it is also worth mentioning that the schemes in Figure 13a,b do not account for the formation of the V_3_O_7_ phase, which is absent in the compact films and only appears in the nanocolumnar cases whenever VO_2_ is formed. This means that the formation of V_3_O_7_ must be triggered not only by the migration of oxygen in the film network, but also by the presence of pores/defects and the swelling of the film upon oxidation.

Based on the results above, it is demonstrated that the use of nanocolumnar films provides several key advantages, in comparison with compact films, when subjected to a low-temperature oxidation process to form VO_2_ crystallites, such as the following: (i) the nanocolumnar films possess a much higher specific surface that makes the adsorption of oxygen much more efficient (promoting the formation of a larger amount of V_2_O_5_ and VO_2_ and the removal of a larger amount of undesired opaque amorphous VO_x_) and (ii) the possibility to accommodate the swelling of the material into the structure thanks to the existence of large empty voids that serve to release stress and that would cause the direct delamination of the coating should it be compact. Finally, it is noteworthy that the features of the film nano-VO_1.5_ (280 °C) were stable for more than one year, depicting the same modulation capabilities and structural features as just after its fabrication, despite the fact that it was exposed to the atmosphere, experiencing variations in environmental temperature during all this time, and that it was not protected by any interfacial encapsulation or by any other means [58]. This implies that both the formation of V_2_O_5_/VO_2_ on the accessible surface of the film and the swelling of the material are enough to limit the diffusion of oxygen and avoid the eventual transformation of the film into the non-thermochromic highest oxidized phase (V_2_O_5_) even after such a long time. Moreover, it also means that the sample is stable enough to withstand changes in environmental temperature (between ~20 °C and ~40 °C).

## 4. Conclusions

From the previous results and discussion, the first novel conclusion of this work is that there is a window of conditions to prepare VO_2_-containing films at temperatures below 300 °C. The conditions found require the use of porous VO_x_ films as a precursor and a precise control of both the porosity of the films and the [O]/[V] ratio prior to the oxidation. In this regard, the use of the well-established MS-OAD technique has proven to be quite straightforward for the precise control of these features. In particular, the feasibility of producing VO_2_ at relatively low temperatures (280 °C) using industrially scalable methods, such as MS-OAD, is demonstrated by the low-temperature oxidation of an amorphous nanocolumnar VO_x_ film. Furthermore, the fact that similar results can be obtained using either pure oxygen or an air flow in the low-temperature oxidation process suggests that the purity of the oxidizing atmosphere is not critical to achieve the thermochromic property at low temperatures. This evidence, together with the widespread use of the magnetron sputtering technique in the industry, supports the use of the proposed novel method as a basis to fabricate thermochromic VO_2_ films at temperatures below 300 °C in mass production facilities.

In this work we have obtained final films with variable transparency and thermochromic capabilities. In particular, we have produced a relatively good thermochromic response as proved by the achievement of optimum values of $T_{lum}$ around 25% (a parameter that is usually employed as an indicator of luminosity for non-doped VO_2_ films) and a $∆T_{sol}$ of 12.5%, this latter taken as a reference of the radiation modulation features. For these samples, resistivity vs. temperature also exhibited a hysteresis loop with a variation in the electrical properties of more than two orders of magnitude. Hall effect measurements revealed the n-type nature of the films with a reverse evolution of electron concentration and mobility, resulting in abrupt changes in resistivity at the transition temperature that have been mainly assigned to electron concentration changes. Based on a thorough characterization analysis of the films using Raman, XRD, RBS/NRA, and electron microscopy tools, a model to explain the main quantities mediating the low-temperature formation of VO_2_ has been proposed. Basically, this takes into account the swelling of the VO_x_ structure upon the incorporation of oxygen into the network, the low-temperature formation of V_2_O_5_ and VO_2_ crystallites on the surface of the film in contact with the oxygen atmosphere, and the limitation of the oxygen diffusion towards the inside of the material due to the formation of these phases. In this way, the precise control of the initial porosity of the sputtered films and their actual [O]/[V] ratio have resulted in being key for a precise control of the final film morphology and thermochromic modulation properties.

## Figures and Tables

**Table 1:** List of samples analyzed in this paper and deposition conditions.

Sample	Power (W)	Deposition Time (min)	O_2_ Flow (sccm)	Substrate Tilt Angle (°)	[O]/[V] (±0.15)
nano-VO_1.9_	150	105	0.2	85	1.9
nano-VO_1.7_	200	93	0.2	85	1.7
nano-VO_1.5_	225	82	0.2	85	1.5
nano-VO_1.4_	250	75	0.2	85	1.4
nano-VO_1.3_	275	68	0.2	85	1.3
compact-V	150	60	0	0	0
compact-VO_0.4_	150	60	0.2	0	0.4
compact-VO_0.5_	150	60	0.5	0	0.5
compact-VO_0.9_	150	60	0.7	0	0.9
compact-VO_1.5_	150	60	1.0	0	1.5

## Data Availability

The original contributions presented in this study are included in the article. Further inquiries can be directed to the corresponding authors.
